# Reasons Why the Profession Should Labor to Diffuse a General Knowledge of Medical Science among the People

**Published:** 1869-08

**Authors:** 


### Miscellaneous.

### Reasons why the Profession should labor to diffuse a general knowledge of Medical Science among the People.

The duty of the profession to diffuse a knowledge of medical science among the people is rendered imperative by these *four* considerations:

1. *Because all people everywhere need, and should have, some general knowledge of the human body in health and disease.*

The time has gone by when it was thought to be necessary that learning should be confined to the few. It is the glory of our century that knowledge of all kinds is diffused among the masses of the people. The time was when theology was confined to the clergy and was the privilege of monks and cloisters; religion is now the duty and the joy of the ignorant and the lowly. The time was when all government and law were in the hands of a few aristocrats, and even of some single monarch; in our day and country the people rule, the kings and queens, presidents and senators, are but their servants.

Science must now follow in the wake of theology and government. If the masses of the people are to have all the power in Church and State, they certainly must not be left in ignorance. For the ignorance of humanity there is only one antidote, and that is knowledge. Of all departments of knowledge, none is so important as that of ourselves. It is impossible to know ourselves without knowing the structure of the human body, the functions of its organs, and the laws of health. It is impossible to acquire this knowledge without careful study, diligent reading and patient repetition, in all the recognized methods of imparting knowledge. It must be taught to children in the school and by the domestic fireside, and in juvenile literature. It must be taught to parents from the pulpit, the platform, and in the periodical press, and in such works as these.

The present ignorance of society in regard to anatomy, physiology, and the laws of health are truly appalling. Even the clergy, who are so advanced in general culture, and who should be the teachers of hygiene as a part of morality, are as a profession utterly in the dark in regard to the simplest laws of life and health. Theologians and professors, college presidents and pulpit orators, who have learned all important languages, living and dead, who can repeat at call all the imbecile and insane kings of Europe and the dates of their administrations, do not even suspect the nature of the processes of respiration or of digestion every moment going on in their own bodies; and even give no reason for the faith that is in them, that the brain rather than the liver is the organ of the mind.

Even men of general science, who plan great inventions and understand all the machinery of man's devising, know nothing of the most wonderful machine of all—the human body. If these things be done in the green tree, what shall be done in the dry? If our teachers, and the teachers of our teachers, know little or nothing of themselves, what shall we say of the great masses of the people? What shall we say of the millions of farmers, mechanics, laborers, and the solid yeomanry of our land, on whose virtue and intelligence the welfare of the republic must ever depend?

*The profession must exert its influence to introduce the systematic study of hygienic science in all of our colleges and institutions of learning.*

Time was when the standard of scolarship was necessarily estimated by the extent of one's familiarity with dead languages; when the span of a thousand years—the dark ages of humanity—intervened between the scholar in the cloister and the literary wealth of the world; when, in short, the student was forced to choose between treasuring up the learning of ancient times, and knowing nothing at all.

That necessity has long since gone by, but the system of instruction to which it gave rise in its leading features lives to-day. Bacon never uttered a profounder or more beautiful thought than when he said what was called the antiquity of the world is really its youth. If the ancients could be alive again to-day, they surely would be the first to bow at the feet of the nineteenth century.

When we consider the marvellous scientific progress of the last century—that within that time Geology has arisen out of the darkness of conjecture and has developed into a more comprehensive and enduring science; and that by the discovery of hydrogen by Cavendish, of oxygen by Priestley, of nitrogen by Rutherford, and by the labors of Sir Humphrey Davy, Liebig, and their followers, the science of chemistry has been as it were created, and all since the year 1766; that within less than this time that universal agent, Electricity, has revealed itself to man in its effects if not in its nature—has indeed deigned to serve him as his fleetest messenger through the air and under the sea, as the faithful and rapid copyist of works of art, as a powerful means of illumination, and as a most effective healer in disease; that within the last fifty years the mechanic arts, in their myriad ramifications, have made more effective progress than other eras have witnessed in twice as many decades; when we consider that astronomy, the most ancient of sciences, the boast of the Egyptians and Chaldeans—which is indeed in its very essence a study of centuries—has not been without its refinements even during the present generation—nay, even within the year that is just passed; when, I say, we thus consider all that the last fifty years has done for science—and more than all, when we contemplate the wondrous possibilities of the fifty years to come, and for which we now have but laid the foundation; and when, on the other hand, we consider how little these branches are taught or even suggested to our undergraduates, we can but wonder that an age which has revolutionized society by its activity in science, has made so little impression on those institutions that ought to be, if they are not, the centres and the repositories of the world's progress.

It is neither necessary nor desirable that hygienic or other science should supplant the languages. It is the duty of the profession, however, to see that in all our institutions of learning it is placed on the *same footing as all other important departments;* that it receives something more than a merely incidental and superficial attention; and that is made *equally binding with all other recognized studies of the course.*

2. *Because physicians are the only class who are authorities in medical science, and who are qualified to give instruction in it.*

Medical science is a large subject, and it takes a lifetime to comprehend it. People look to those whose lives are devoted to this subject to teach them what they aught to know. They have a right to do so. Of those to whom much has been given, much will be required. If we know that which will be of service to our fellows, we have no right to keep it to ourselves alone. A miser of knowledge is even more censurable than a miser of money, because he is more intelligent, and therefore more responsible. It is even more wrong for us to hoard knowledge than to hoard specie, for knowledge is more valuable than gold or silver or precious stones.

Until quite recently the clergy have been the chief instructors of the people in medical science; but they have unfortunately taught more of error than of truth. The fault, however, is not with the clergy but with the physicians. The clergyman must be himself instructed before he can instruct others. The duty of teaching medical science to the clergy devolves upon the physician, because in all such matters he is the first authority and last appeal. It is right and proper and noble for the pastor to teach his flock the laws of health, and to enjoin their observance as a high moral duty; but he must know whereof he affirms, and the true knowledge on these themes he must learn from the physician.

3. *Because the instruction of the people in medical science has been almost entirely in the hands of ignorant and unprincipled charlatans.*

This lamentable and well-known fact, which ought long since to have aroused the profession to its great duty, seems to have had the opposite effect, and has deterred them from attempting any systematic instruction of the people. There are those even now who fear to write or lecture for the masses, lest they may thereby become classed with the ignorant and villainous quacks who in this country have appropriated this department almost entirely to themselves. I hold to a very different doctrine. I hold that the example of charlatans, so far from discouraging, should rather stimulate the profession to follow after them and drive them off the track. It is because the enemy have planted tares in the field, that we should enter in and sow the good seed. It is because the philistines have already invaded the land, that we should hasten to take possession.

The noblest and best part of our mission is not to cure disease, but to prevent it. The true and only way to prevent disease is to diffuse through all ranks of society a general knowledge of the human body and of the laws of health.

There may be those who fear lest the profession may lose its dignity by coming down from its lofty eminence and feeding the hungry multitude. In the infancy of science, in the darkness of the middle ages, such fear was, perhaps, not unnatural; but the time for that has now long gone by. When the sun is rising it gilds only the higher mountain tops; when it mounts to noonday it sends its rays, bright, warm, and abundant, into the depths of the valleys and the darkest crevices the rocks. Just so when science was first rising upon the world, its light was only seen and its warmth only felt by the philosopher, the recluse; as it is now ascending higher in the sky, it should shine, with wisdom and healing in its beams, on the walks of the humble, the lowly and the sorrowing.

Science is no more degraded by ministering to the wants of the people than is the sunlight when it trails its beams along the valleys; or the rain when it falls alike on the evil and the good.

Jean Paul Richter has somewhere presented in substance this simile, which the disciple of science should ever bear in mind: "Beautiful is the eagle when it soars aloft in the sky and plumes its distant flight towards the sun, but more beautiful still when it descends to the earth and brings food to its helpless offspring in their nest; so the philosopher is noble when he lives above the world in the cold atmosphere of science, but nobler still when he descends from his lofty heights and brings hope and comfort to the suffering sons of men."

4. *Because the profession will elevate and benefit itself by thus instructing the people in medical science.*

All physicians the world over will agree that ignorant people make the worst patients. The lower classes are proverbially exacting and unreasonable, and too often unappreciative. In proportion as people are educated—and especially in science—in that proportion do they become considerate towards their physician, obedient to his orders, and grateful for his services.

The effect of the popularization of medical science will be not to diminish the practice of the profession, but to increase it. Patients are deterred from consulting educated physicians, not by knowledge but by ignorance; not by their ability to prevent or treat diseases, but by their inability to distinguish between those conditions which are beyond all hope, and those which in scientific hands are both relievable and curable.

When the people are educated to a full understanding of the wonderful achievements of science in the past, and the vast progress that it is making in the present, and the wide distinction between the physician and the quack, then will they know—what the masses of our country have yet to learn—that the educated members of the profession are not the enemies but the friends of advancement, and that on the average they are as much more successful than the charlatans, as they are more scholarly and more honest.

It is only by a general diffusion of popular science that the vast army of charlatans—that are now working such ruinous havoc in the best ranks of the society—can be successfully combated and dispersed. The scientific man is pained to his heart's core when he sees—as every day he is compelled to see—the best educated and finest cultured minds of the country—our leaders in literary, professional and business life—ruined in health and in purse by the vilest quacks that ever disgraced any age or country. The quackery of our day feeds and fattens on the ignorance of the learned. It derives its rich support from the fact that the people know all other things better than they know science. The scientific physician who long gazes upon this great rush of humanity after quack doctors, quack books, quack medicines—after all forms of error and one-sided "pathies" and "isms," feels much like the philanthropist who, from the bank of a mighty stream, sees his fellow-beings hurried along in the flood and ingulfed in the whirlpool, while he is powerless to save.

Salvation from quackery will only come from popular instruction.

Besides all this, it is the duty of the profession, through the popularization of science, to make itself a *power in society.*

It is our duty in this way to make our influence more widely felt as a ruling force through all the departments of modern activity.

For all these reasons we hail with joy the recent endeavors of some of our leading physicians to popularize medical science. The system of lectures on science lately attempted by Professors Huxley and Carpenter in London; the noble example of Professors Willard Parker and E. R. Peaslee in New York; the recently published essays of Bowditch, Jarvis, Allen, Youmans, Hammond, Flint, Mitchell, Griscom, Peters, Roosa, Harris, Ryford, and other leading authori ies in our profession; and the magnificent and useful treatise of our eminent countryman, Prof. John C. Dalton—all these are the emphatic protests on the part of the profession that the people shall no longer dwell in darkness, that the medieval age of narrow and selfish exclusiveness has passed away, and that men of science shall hereafter follow in the path of theologians and law-givers, and sow the good seed of truth broadcast through society.

In order to popularize science it is not enough to provide text-books for the young. We must sow beside all waters. We must make the magazines, the daily and weekly press, the platform, the lecture hall, the organizations of philanthropy, the pulpit and the Sabbath-school, channels of communication, through which knowledge of science shall flow to the uttermost corners of the earth.—*Home Physician.*

